# Expression and clinical relevance of epithelial and mesenchymal markers in circulating tumor cells from colorectal cancer

**DOI:** 10.18632/oncotarget.14065

**Published:** 2016-12-21

**Authors:** Ren Zhao, Zhen Cai, Sheng Li, Yong Cheng, Hua Gao, Fang Liu, Shiyang Wu, Suyan Liu, Yan Dong, Lei Zheng, Wenbin Zhang, Xiaojun Wu, Xueqing Yao

**Affiliations:** ^1^ Department of General Surgery, Ruijin Hospital, Shanghai Jiaotong University School of Medicine, Shanghai, China; ^2^ Department of Laboratory Medicine, Sino-UK Circulating Biomarkers’ Exploration and Detection Center, Nanfang Hospital, Southern Medical University, Guangzhou, China; ^3^ 3Department of General surgery, Guangdong General Hospital & Guangdong Academy of Medical Sciences, Southern Medical University, Guangzhou, China; ^4^ Department of Gastrointestinal Surgery, First Affiliated Hospital of Chongqing Medical University, Chongqing, China; ^5^ Department of General Surgery, First Affiliated Hospital of Xinjiang Medical University, Urumqi, China; ^6^ SurExam Bio-Tech Co., Guangzhou, China; ^7^ Department of Gastrointestinal Surgery, First Affiliated Hospital of Xinjiang Medical University, Urumqi, China; ^8^ Department of Colorectal Surgery, Sun Yat-sen University Cancer Center, Guangzhou, China

**Keywords:** colorectal cancer, circulating tumor cells, epithelial-mesenchymal transition, clinical stage, metastasis

## Abstract

Circulating tumor cells (CTCs) with phenotypic hallmarks of epithelial-mesenchymal transition (EMT) reportedly contribute to tumor metastasis in different cancer types. We therefore evaluated the expression of EMT markers in CTCs obtained from a large cohort of Chinese patients with colorectal cancer (CRC) and investigated their clinical relevance. The CanPatrol^TM^ CTC enrichment technique was used to isolate and classify CTCs. CTCs were detected in 1046 of 1203 patients (86.9%), and three phenotypes were identified based on the expression of epithelial and mesenchymal markers: epithelial CTCs, biophenotypic (epithelial/mesenchymal) CTCs, and mesenchymal CTCs. Total CTC numbers positively correlated with both clinical stage and lymph node metastasis and distant metastasis. Furthermore, both biophenotypic and mesenchymal, but not epithelial, CTCs, correlated with the above parameters, suggesting CTCs displaying a mesenchymal phenotype denote more aggressive disease and metastatic potential. This is the first study to demonstrate a significant correlation between CTCs displaying a mesenchymal phenotype and both clinical stage and metastasis in a large cohort of patients with CRC. Our findings suggest that assessment of not only epithelial, but also mesenchymal markers in CTC analyses may offer valuable assistance for tumor staging and metastasis evaluation in patients with CRC.

## INTRODUCTION

Colorectal cancer (CRC) is one of the most common cancers worldwide, with approximately 1,400,000 new cases and 693,900 deaths reported in 2012 [1]. At initial diagnosis about one-fourth of CRC patients present with metastases, which will eventually affect up to half of the patients, contributing to CRC's high mortality rates [2]. Although early prediction and diagnosis of metastasis could have important implications on patient management in CRC, current diagnostic methods are usually unable to provide early information about ongoing metastasis and to accurately predict their occurrence and outcomes [3]. New diagnostic methods are therefore eagerly required to address these issues and to provide real-time information on therapy efficacy.

The presence of circulating tumor cells (CTCs), a small population of cancer cells in the peripheral blood that have detached from a primary or metastatic tumor, has been evaluated in various cancers, including CRC [4]. The isolation and characterization of CTCs through “liquid biopsy” methods has a potentially high prognostic significance and may also serve to monitor treatment efficacy in CRC and other cancers. CTCs have been detected in all stages of CRC [5]. In early stage CRC, CTC assessment may help select high-risk patient candidates for adjuvant chemotherapy [6]. In primary or non-metastatic CRC, detection of CTCs is indicative of poor prognosis [7, 8]. Similarly, in advanced or metastatic CRC, the presence of CTCs has been associated with disease progression and poor outcomes [9–14]. During treatment, CTCs may act as a surrogate biomarker to guide treatment selection and assess treatment benefit [10, 13, 14]. Additionally, molecular analyses of CTCs may aid in the prediction of drug resistance and the selection of anticancer drugs [15, 16].

Besides their predictive and prognostic relevance, CTCs are considered as main sources of tumor metastases [17], and are thus emerging as a novel target for early metastasis detection [18]. Aberrant activation of the epithelial-mesenchymal transition (EMT) program has been implicated in the dissemination of CTCs. EMT endows CTCs with mesenchymal and stemness phenotypes, and is an early event in the metastatic process [17, 19]. It is thus conceivable that detection of EMT markers in CTCs may facilitate the early detection of metastases as well as the assessment of new drugs in clinical trials.

Research studies reporting on the expression of EMT markers in CTCs have been conducted mainly in patients with breast, prostate, liver, and lung cancer [20]. Epithelial cell adhesion molecule (EpCAM) and cytokeratins (CKs) are usually used as markers to identify epithelial CTCs, while vimentin (VIM), TWIST1, AKT2 and SNAI1 are commonly used to identify mesenchymal CTCs [20]. EpCAM is a cell surface glycoprotein that mediates cell-cell adhesion in epithelial tissues [21]; diminished EpCAM expression is linked to tumor invasiveness and progression in CRC [22]. CKs are members of the intermediate filament (IF) protein family found in the cytoskeleton of epithelial cells [23] and are useful markers of metastasis onset in CRC [23]. VIM, a major component of the IF family of proteins, is ubiquitously expressed in mesenchymal cells, and its overexpression in CRC cells correlates with increased migration and invasive potential [24]. TWIST1 is a helix-loop-helix protein involved in embryogenesis. Its reactivation in cancers leads to EMT, and its dysfunction contributes to tumor development and progression [25]. In CRC, TWIST1 expression was found to be restricted to tumor cells and correlated with lymph node metastasis and poor prognosis [26]. AKT2, a member of the AKT kinase family, is frequently upregulated in various cancers, including CRC [27], where it contributes to the EMT process via the phosphatidylinositol 3′ kinase (PI3K)/AKT pathway [28]. In CRC cells, overexpression of AKT2 led to the formation of micrometastases [27]. SNAI1 (also known as Snail) is a zinc-finger transcription factor that mediates EMT in several tumor types, and its overexpression in human CRC cells enhances invasiveness and metastatic behavior [29].

Several studies highlighted the importance of the mesenchymal phenotype in CTCs during tumor metastasis and progression. Min and colleagues showed that Snail expression in CTCs may be associated with extra-hepatic metastasis of hepatocellular carcinoma (HCC) [30]. A study by Li et al. provided evidence that co-expression of TWIST and VIM in CTCs was highly correlated with portal vein tumor thrombus, and suggested that the detection of both TWIST and VIM in CTCs could predict HCC metastasis more accurately [31]. Yu et al. reported that CTCs exhibit dynamic changes in epithelial and mesenchymal composition, and that mesenchymal CTCs are related to metastasis and resistance to chemotherapy in breast cancer [32]. In addition, there is an emerging notion that the hybrid epithelial/mesenchymal phenotype, characterized by the partial loss of cell-cell adhesion and gradual acquisition of migratory and invasive traits during the EMT process [33], is associated with aggressive tumor progression [34, 35].

However, owing to the technical challenges and high costs associated with CTC analyses, the sample size in most EMT CTC-related studies is generally small, and thus the clinical significance of EMT phenotypes in CTCs remains to be systematically analyzed. Moreover, only a few studies assessed the presence of EMT in CTCs from CRC and addressed its clinical relevance. For instance, a recent study established cell-surface vimentin as a universal marker to examine EMT in CTCs from patients with metastatic colon cancer, showing a correlation between the number of EMT CTCs and therapeutic outcome [36]. However, these results should be interpreted with caution due to the limited sample size and the use of a single mesenchymal marker. By contrast, large-scale analyses of EMT phenotypes in CTCs, using a combination of multiple epithelial and mesenchymal markers such as EpCAM, CK8/18/19, VIM, TWIST1, AKT2, and SNAI1, could provide more convincing evidence of the clinical value of different CTC phenotypes, and expand our understanding of their contribution to the development and progression of cancer.

This study was designed on the premise that a large-scale analysis of EMT markers in CTCs from patients with CRC would provide a strong and reliable tool to assess the correlation between the different phenotypic hallmarks of CTCs and patient characteristics, such as clinical stage and metastatic status. To this end, we adopted the CanPatrol^TM^ CTC technique (SurExam, Guangzhou, China) [37], which employs a filter-based CTC capture method followed by RNA in situ hybridization (ISH), to evaluate the feasibility of using multiple epithelial (EpCAM and CK8/18/19) and mesenchymal (VIM, TWIST1, AKT2 and SNAI1) markers to classify CTCs in a large cohort (*n* = 1203) of Chinese patients with CRC. We expect that our study, which explores the correlation of distinct phenotypes of CTCs with clinical stage and metastatic status, will provide a reliable reference to assist implementing this technology in the clinical setting.

## RESULTS

### Expression of epithelial and mesenchymal markers in DLD-1 cells and leukocytes from healthy blood donors

**Figure 1:**
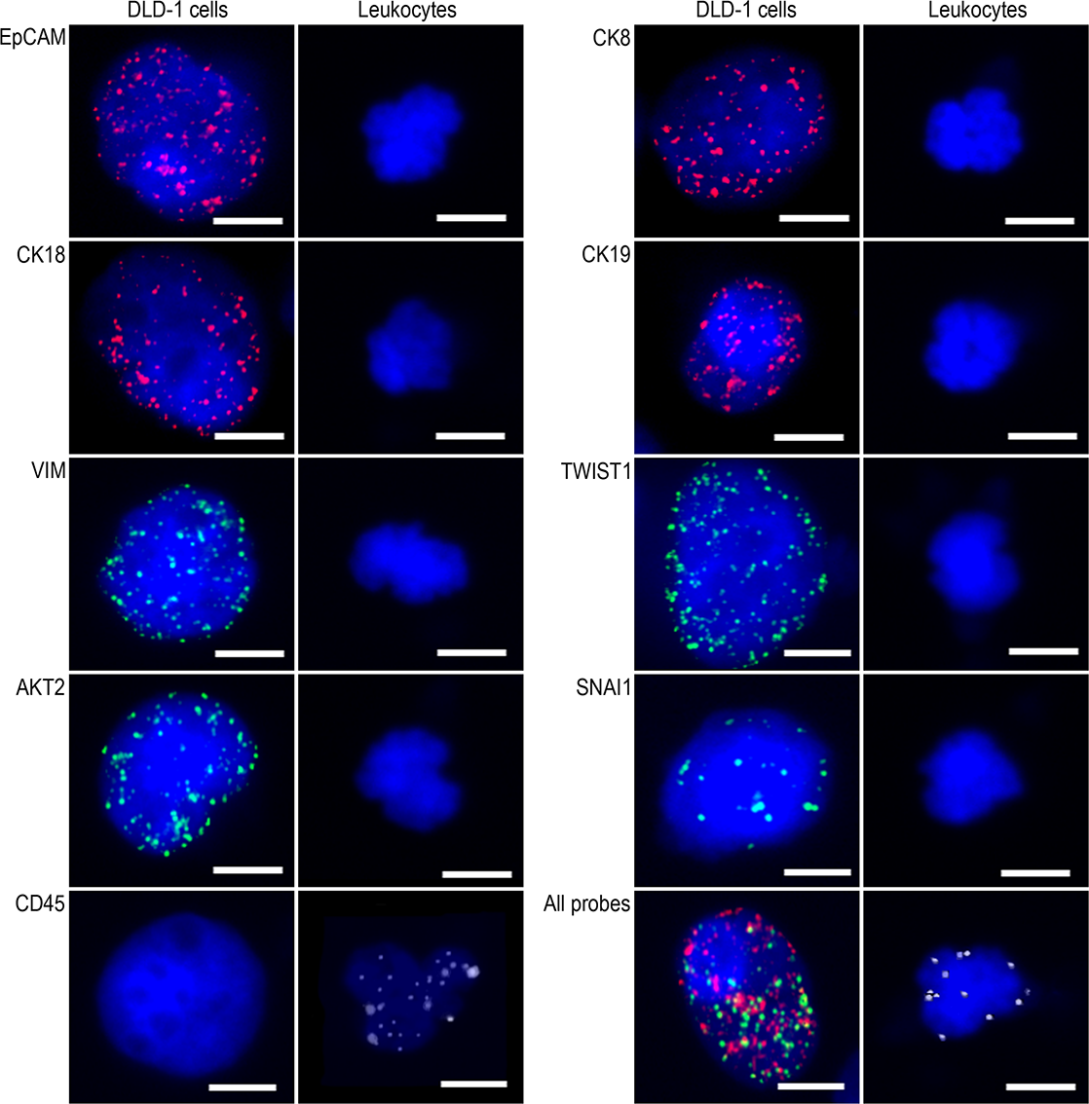
Epithelial and mesenchymal marker expression in DLD-1 cells and leukocytes Representative microscopic images of fluorescent RNA-ISH detection of EpCAM, CK8/18/19, VIM, TWIST1, AKT2, and SNAI1 expression. Bars = 5 μm.

To analyze the feasibility of characterizing CTCs by the combined detection of multiple epithelial (EpCAM and CK8/18/19) and mesenchymal (VIM, TWIST1, AKT2 and SNAI1) markers, DLD-1 colorectal adenocarcinoma cells were spiked into 5 ml of blood from healthy donors. In situ RNA hybridization detection showed that EpCAM, CK8/18/19, VIM, TWIST1, AKT2, and SNAI1 were expressed in DLD-1 cells but not in leukocytes, whereas CD45 was expressed in leukocytes but not in DLD-1 cells (Figure 1). These results suggest that the combined use of EpCAM, CK8/18/19, VIM, TWIST1, AKT2, and SNAI1 is a valid strategy for CTC classification.

### Validation of the CTC classification protocol in CRC blood samples

**Figure 2:**
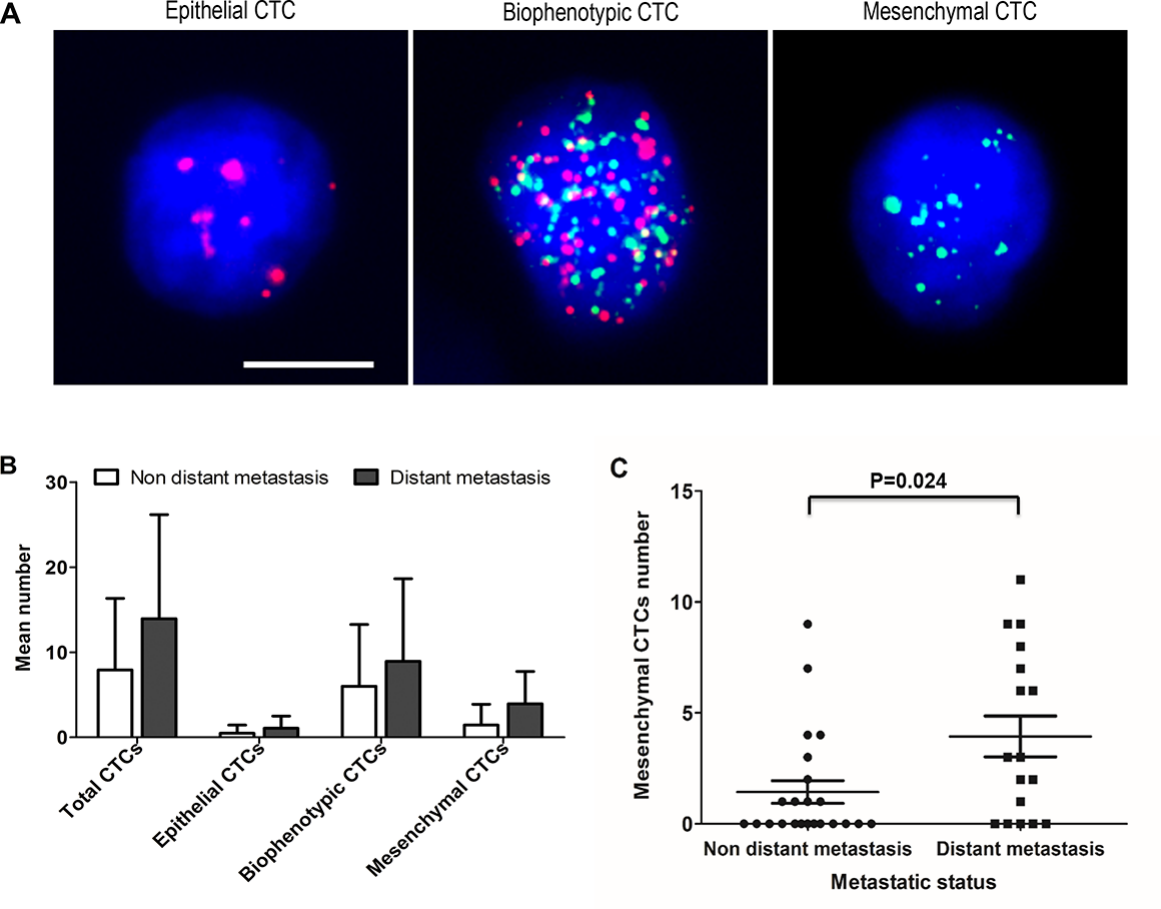
Assessment of CTC phenotypes in blood samples from patients with CRC (**A**) Representative images of the three CTC phenotypes. Epithelial biomarkers are indicated by red dots; mesenchymal biomarkers are indicated by green dots. Cells with red dots represent epithelial CTCs, cells with green dots represent mesenchymal CTCs, and cells with red and green dots represent biophenotypic CTCs. Bars = 5 μm. (**B**) Mean numbers of total CTCs, epithelial CTCs, biophenotypic CTCs, and mesenchymal CTCs according to metastatic status. (**C**) Correlation between mesenchymal CTCs and distant metastasis.

The CTC detection method described above was further tested in blood samples from 40 patients with CRC. These included 23 patients without distant metastasis (9 patients at stage I, 6 at stage II, and 8 at stage III), and 17 patients with verified distant metastasis (all at stage IV). CTCs (≥ 1 per 5 ml blood) were detected in 36/40 (90.0%) CRC patients, and three CTC phenotypes, namely epithelial, biophenotypic, and mesenchymal, were identified (Figure 2A).

CTCs were detectable in 20/23 (87.0%) patients without distant metastasis and in 16/17 (94.1%) patients with distant metastasis. The average total CTC number was increased in the distant metastatic stages of CRC compared with earlier stages without distant metastatic disease (13.94 vs. 7.91, respectively; Figure 2B). The mean number of biophenotypic and mesenchymal CTCs in the distant metastatic stages (8.94 and 3.94, respectively; Figure 2B) was also increased compared with the early stages (6.00 and 1.43, respectively; Figure 2B). Furthermore, Spearman's rank correlation analysis revealed that the number of mesenchymal CTCs was related to distant metastasis (*P* = 0.024) (Figure 2C), whereas no correlation was observed between total, epithelial or biophenotypic CTCs numbers and metastasis. These results indicate that it is feasible to use EpCAM, CK8/18/19, VIM, TWIST1, AKT2, and SNAI1 for EMT-based CTC classification in patients with CRC.

### Large-scale analysis of epithelial and mesenchymal markers in CTCs from CRC patients

Peripheral blood samples were collected from an additional 1163 patients with CRC for CTC isolation and characterization. In total, 1203 patients with CRC of any stage were included in the study. Among these, 249 presented lymph node metastasis and 303 had verified distant metastasis. Characteristics of the study population and CTCs prevalence by subgroups are summarized in Table 1. CTCs (≥ 1 per 5 ml blood) were detected in 1046/1203 patients (86.9%). The representation of the epithelial, biophenotypic, and mesenchymal CTC phenotypes was 40.0% (481/1203), 76.8% (924/1203) and 56.9% (684/1203), respectively (Table 1). Total and phenotype-specific CTC numbers for the 1203 CRC patients are summarized in Table 2. For all patients, total CTC mean number was 7.80 (range: 0 to 125). The average number of epithelial, biophenotypic and mesenchymal CTCs was 1.32 (range: 0 to 74), 4.51 (range: 0 to 86), and 1.98 (range: 0 to 40), respectively.

**Table 1:** Characteristics of the study population and CTCs prevalence according to different subgroups

	Number of patients, *N* (%)				
	Patients	CTC-positive	Epithelial CTCs	Biophenotypic CTCs	Mesenchymal CTCs
Total	1203 (100.0)	1046 (86.9)	481 (40.0)	924 (76.8)	684 (56.9)
Clinical stage					
I	213 (17.7)	174 (81.7)	83 (39.0)	143 (67.1)	95 (44.6)
II	438 (36.4)	361 (82.4)	168 (38.4)	318 (72.6)	203 (46.3)
III	249 (20.7)	228 (91.6)	108 (43.4)	209 (83.9)	167 (67.1)
IV	303 (25.2)	283 (93.4)	122 (40.3)	254 (83.8)	219 (72.3)
Metastasis					
No	651 (54.1)	535 (82.2)	251 (38.6)	461 (70.8)	298 (45.8)
Lymph node metastasis	249 (20.7)	228 (91.6)	108 (43.4)	209 (83.9)	167 (67.1)
Distant metastasis	303 (25.2)	283 (93.4)	122 (40.3)	254 (83.8)	219 (72.3)

**Table 2:** Total and phenotype-specific CTC numbers according to different subgroups in CRC

	Total CTCs		Epithelial CTCs		Biophenotypic CTCs		Mesenchymal CTCs	
	Range	Average	Range	Average	Range	Average	Range	Average
Total	0∼125	7.80	0∼74	1.32	0∼86	4.51	0∼40	1.98
Clinical stage								
I	0∼57	5.70	0∼49	1.63	0∼40	2.72	0∼40	1.35
II	0∼125	6.56	0∼74	1.33	0∼86	3.91	0∼39	1.32
III	0∼93	8.59	0∼25	1.35	0∼59	4.86	0∼31	2.38
IV	0∼84	10.42	0∼16	1.04	0∼59	6.34	0∼35	3.04
Metastasis								
No	0∼125	6.28	0∼74	1.43	0∼86	3.52	0∼40	1.33
Lymph node metastasis	0∼93	8.59	0∼25	1.35	0∼59	4.86	0∼31	2.38
Distant Metastasis	0∼84	10.42	0∼16	1.04	0∼59	6.34	0∼35	3.04

After excluding CTC-negative patients, the epithelial, biophenotypic, and mesenchymal CTC average ratios in each CTC-positive patient were 19.2%, 55.0%, and 25.8%, respectively. These results show that biophenotypic CTCs accounted for most CTCs, with a significantly higher average ratio than both epithelial and mesenchymal CTCs (*P* = 0.000 in both cases).

### Correlation between CTC phenotype and CRC clinical stage

**Figure 3:**
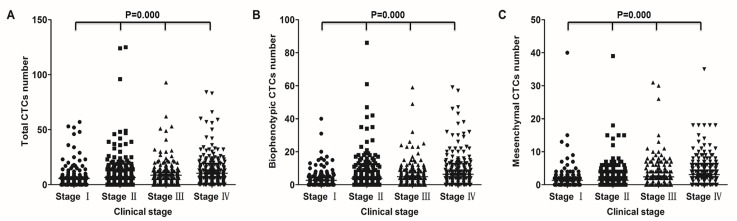
Correlation of CTCs with clinical stage in CRC patients Correlation between total (**A**) biophenotypic (**B**) and mesenchymal (**C**) CTCs and clinical stage.

Upon stratification by clinical stage, CTCs were detected in blood samples from 81.7% of patients with CRC stage I, 82.4% with stage II, 91.6% with stage III, and 93.4% with stage IV (Table 1). The average number of CTCs per CRC disease stage was 5.70 (stage I), 6.56 (stage II), 8.59 (stage III), and 10.42 (stage IV) (Table 2). Consistent with the above results, Spearman's rank correlation analysis further revealed that both presence and number of CTCs were related to CRC clinical stage (*P* = 0.000 in both cases; Figure 3A), with higher CTC positive rate and higher number of CTCs observed in the later stages of CRC.

The distribution of each CTC phenotype was also assessed with respect to clinical stage. The detection rate of epithelial CTCs was 39.0% in stage I, 38.4% in stage II, 43.4% in stage III, and 40.3% in stage IV CRC patients (Table 1); the average number of epithelial CTCs for every stage was, respectively, 1.63, 1.33, 1.35, and 1.04 (Table 2). Biophenotypic CTCs were observed in 67.1% of stage I, 72.6% of stage II, 83.9% of stage III, and 83.8% of stage IV patients (Table 1); the average biophenotypic CTCs number was, respectively, 2.72, 3.91, 4.86, and 6.34 (Table 2). The positive rate of mesenchymal CTCs was 44.6% in stage I, 46.3% in stage II, 67.1% in stage III and 72.3% in stage IV CRC (Table 1); the average number of mesenchymal CTCs was, respectively, 1.35, 1.32, 2.38, and 3.04 (Table 2). These results show that both detection rate and mean number of biophenotypic CTCs and mesenchymal CTCs are increased in the later stages of CRC.

Spearman's rank correlation analysis confirmed that these two CTC phenotypes were correlated with disease stage (Figure 3B and 3C; *P* = 0.000), namely, patients in the later stages of CRC were more likely to have increased numbers of both biophenotypic and mesenchymal CTCs. However, no correlation was observed between epithelial CTCs and clinical stage. These results suggest that the presence of CTCs displaying a mesenchymal phenotype correlates with disease severity.

### Correlation between CTC phenotype and CRC metastasis

Based on metastatic status, the positive rate of total CTCs was 82.2% in patients with non-metastatic CRC, 91.6% in patients with lymph node metastasis, and 93.4% in patients with distant metastasis (Table 1); the average number of CTCs was, respectively, 6.28, 8.59, and 10.42 (Table 2). Epithelial CTCs were detectable in 38.6% of patients without metastasis, 43.4% of patients with lymph node metastasis, and 40.3% of patients with distant metastasis (Table 1); the average number of epithelial CTCs was, respectively, 1.43, 1.35, and 1.04 (Table 2). Biophenotypic CTCs were observed in 70.8% of patients without metastasis, in 83.9% of patients with lymph node metastasis, and in 83.8% of patients with distant metastasis (Table 1); the respective biophenotypic CTCs mean numbers were 3.52, 4.86, and 6.34 (Table 2). For mesenchymal CTCs the positive detection rate was 45.8% in non-metastatic CRC, 67.1% for patients with lymph node metastasis, and 72.3% for patients with distant metastasis (Table 1); the average number of mesenchymal CTCs was, respectively, 1.33, 2.38, and 3.04 (Table 2).

**Figure 4:**
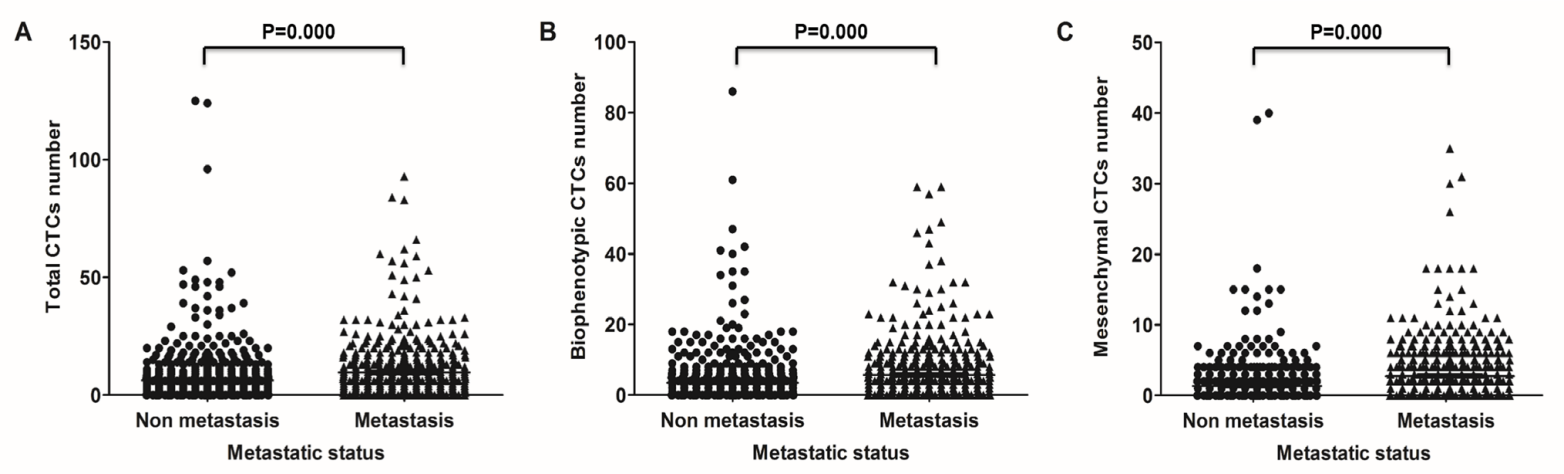
Correlation of CTCs with metastasis in CRC patients Correlation between total (**A**) biophenotypic (**B**) and mesenchymal (**C**) CTCs and metastasis.

Spearman's rank correlation analysis indicated that total CTC presence and number were both positively correlated with lymph node and distant metastasis (*P* = 0.000 in both cases) (Figure 4A). Specifically, both biophenotypic and mesenchymal, but not epithelial, CTCs were correlated with lymph node metastasis and distant metastasis (*P* = 0.000; Figure 4B and 4C). These results suggest an association between the EMT process in CRC cells and the development of metastases.

## DISCUSSION

Using the CanPatrol^TM^ CTC enrichment technique, and a set of probes for the detection of epithelial (EpCAM, CKs) and mesenchymal (VIM, TWIST1, AKT2, and SNAI1) markers, we studied the presence and phenotypic characteristics of CTCs in 1203 Chinese patients with CRC. To the best of our knowledge, this is the largest patient cohort study applying the CanPatrol^TM^ CTC technique to analyze CTCs phenotypes according to the expression of the above biomarkers, and the first also in evaluating EMT phenotypes of CTCs in such a large cohort of CRC patients. This assessment was undertaken to test our hypothesis that the study of CTC's EMT markers in a large CRC patient population would provide a strong and reliable mean to ascertain the correlation of different CTC phenotypic hallmarks with both tumor stage and metastatic status.

Many techniques and methods have been developed for the isolation and characterization of CTCs in the past decade; however, none of them can be considered as the gold standard for the detection of the entire pool of CTCs [38]. The CellSearch technology by Veridex [5, 9], which employs antibodies against the epithelial proteins EpCAM and CKs for capturing and detecting CTCs, is until now the only FDA-approved CTC detection platform. Nevertheless, CellSearch and various other methods can only detect epithelial CTCs, but not CTCs that have undergone EMT and no longer express epithelial markers [39, 40]. VIM, TWIST1, AKT2, and SNAI1 are expressed in CRC pathological tissues, where they promote tumor metastasis by inducing the EMT process [24–29]. The CanPatrol^TM^ CTC technique combines EpCAM, CKs, VIM, TWIST1, AKT2, and SNAI1, and is able to detect not only epithelial CTCs but also biophenotypic CTCs, which have partially lost their epithelial characteristics, as well as mesenchymal CTCs, which have completely lost their epithelial features.

Using the CanPatrol^TM^ platform, we found that CTCs (≥ 1 per 5 ml blood) could be detected in 86.9% of CRC patients. Our CTC positive rates, up to 80% in early or non-metastatic CRC and up to 90% in advanced or metastatic CRCs, were significantly higher than those detected with the CellSearch platform [5, 9, 41]. The high sensitivity of the CanPatrol^TM^ CTC technique could be basically attributed to two aspects [37]: first, this technique isolates CTCs using an unbiased and simpler filter-based method, thus reducing the loss of CTCs caused by multiple centrifugation and washing steps. Second, it combines multiple epithelial and mesenchymal markers and uses a multiplexed RNA-ISH method to label the isolated CTCs, providing higher sensitivity and background suppression.

Previous studies using the ‘isolation by size of epithelial tumor cells (ISET)’ platform or similar methods have found that CTCs that underwent EMT could be identified in most patients [36, 42], an observation supported by our results. We found that biophenotypic CTCs accounted for most CTCs (55.0%) in all patients, suggesting that biophenotypic CTCs co-expressing both epithelial and mesenchymal markers represent the paradigm of a phenotypical continuum between epithelial and mesenchymal states. In this regard, cells with partial EMT features or possessing hybrid E/M phenotypes have been suggested to have a much large repertoire of survival strategies under many stress conditions [33], which may explain the high proportion of biophenotypic CTCs in our patient cohort. These evidences further imply that the EMT process is important for the formation and dissemination of CTCs.

In this study we demonstrated that total CTCs’ presence and number are positively correlated with CRC disease stage. Further analysis revealed that this was true for biophenotypic and mesenchymal, but not epithelial, CTCs, which suggests that CTCs undergoing EMT or displaying a full mesenchymal phenotype are indicative of more serious disease. Altogether, this evidence strongly suggests that the assessment of CTCs holds great potential as an adjunct for CRC staging [38]. Altogether, these data may be useful to design *in vivo* experiments to underscore the role of CTCs in CRC progression, and to help defining the impact of the diverse CTC phenotypes on CRC staging.

In agreement with the notion of CTCs as potential seeds for metastatic dissemination [17], our results also showed that total CTCs correlated with both lymph node and distant metastasis of CRC. As with clinical stage, sub-analyses by CRC metastatic status demonstrated a significant correlation for biophenotypic and mesenchymal, but not epithelial, CTCs. The correlation between biophenotypic or mesenchymal CTC presence and CRC metastasis detected in our study is in line with previous reports [30–32, 34, 35] and supports the notion that CTCs displaying a mesenchymal phenotype have a strong metastatic potential [43]. In this regard, it is important to note that mesenchymal CTCs showed, among the three phenotypes, the most segregated distribution with respect to metastatic status (non-metastatic CRC: 45.8%; lymph node metastasis: 67.1%; and distant metastasis: 72.3%). These data encourage future studies on the mechanisms by which CTCs initiate metastases, and highlight the potential clinical value of assessing EMT hallmarks in CTCs for the early detection of CRC metastases.

In summary, our study showed, for the first time and in a very large cohort of patients, that the presence of biophenotypic and mesenchymal CTCs, rather than epithelial CTCs, is correlated with CRC disease stage and metastasis. These results suggest that CTCs, especially those displaying a mesenchymal phenotype, have the potential to serve as biomarkers to assist in tumor staging and evaluation of metastasis in patients with CRC. Furthermore, considering the contribution of CTCs to disease development and metastasis formation, our findings may help researchers develop CTC-targeting therapies to improve the prognosis of CRC patients.

Due to the large sample size and the inclusive assessment of epithelial and mesenchymal markers, we believe that our results are robust and valid. Our study has, however, a few limitations. For example, CTC detection was performed only once in each patient, and the clinicopathological features collected were not comprehensive. In this regard, long-term follow-up with new analyses of CTCs and additional clinicopathological records would be essential to understand how the EMT in CTCs could be used to predict metastasis and ultimately improve the outcome of patients with CRC.

## MATERIALS AND METHODS

### Cell culture

The DLD-1 cell line, derived from human colorectal adenocarcinoma, conserves a degree of heterogeneity similar to that of the original tumor, comprising several phenotypes which allow to study tumor heterogeneity and assess the cells’ invasive and metastatic potential [44]. Cells were cultured in RPMI 1640 Medium with 10% fetal bovine serum and 1% penicillin-streptomycin (all from Thermo Fisher, Waltham, USA) at 37°C in an incubator with 5% CO_2_.

### Patients and blood sample collection

Between October 2014 and April 2016, patients with CRC of any stage from hospitals throughout China were enrolled in this study. Main eligibility criteria included pathological diagnosis of CRC, age > 18 years, and no other malignant tumor history or inflammatory disease. Patients were ineligible if they had concurrent solid tumors or inflammatory diseases, or had undergone curative surgical resection and no macroscopic tumor remained. Blood samples were obtained at baseline, i.e., before surgery in patients who underwent curative surgical resection, or before other treatment(s) in patients with palliative resection, or during chemotherapy treatment intervals in patients with advanced disease. Peripheral blood samples (5 ml) were collected by venipuncture from each patient in EDTA tubes and stored at 4°C until isolation of cells (within 4 hours). Peripheral blood samples from healthy donors were used as negative controls or for spiking assays. In all cases, prior written informed consent from each subject and approval from the corresponding Ethics Committees were obtained.

### Isolation and classification of CTCs by the CanPatrol^TM^ CTC technique

Isolation and classification of CTCs was performed using the CanPatrol^TM^ CTC enrichment technique (SurExam, Guangzhou, China), as described in detail recently [37]. Briefly, peripheral blood samples were treated with a red blood cell lysis buffer, and CTCs isolated using a filtration system. A RNA-ISH method was used to enumerate and classify CTCs according to the expression of epithelial and mesenchymal markers. However, in contrast with the previous study describing this method [37] two additional mesenchymal marker probes, AKT2 and SNAI1, were added to further optimize CTC phenotyping. Finally, 4’,6-diamidino-2-phenylindole (DAPI) (Sigma, St. Louis, USA) was used to stain the cell nuclei, and the samples were analyzed with an automated imaging fluorescent microscope (Zeiss, Germany). The red and green fluorescent signals observed in the cells represented epithelial and mesenchymal markers, respectively. A bright white fluorescent signal represented CD45 expression, a marker of leukocytes.

### CTC classification criteria

CTCs were classified into three phenotypes: (1) epithelial marker^+^/mesenchymal marker^-^ /CD45^-^/DAPI^+^ cells (epithelial CTCs); (2) epithelial marker^+^/mesenchymal marker^+^/CD45^-^/DAPI^+^ cells (biophenotypic CTCs); and (3) epithelial marker^-^/mesenchymal marker^+^/CD45^-^/DAPI^+^ cells (mesenchymal CTCs). Leukocytes were identified as CD45^+^/DAPI^+^ cells.

### Statistical analysis

Statistical analysis was performed using SPSS 17.0 software package (SPSS Inc. Chicago, IL). The correlation between two variables was tested using Spearman's rank correlation analysis. All statistical analyses were two-tailed and a *P* value of < 0.05 was considered to be statistically significant.
